# The Role of Loneliness and Ostracism in Adolescents’ Psychological Well‐Being and Substance Use: Family and Teacher Support as Moderators

**DOI:** 10.1002/jad.70067

**Published:** 2025-10-21

**Authors:** Mari Tunkkari, Noona Kiuru, Niina Junttila, Leena Paakkari, Nelli Lyyra

**Affiliations:** ^1^ Department of Psychology University of Jyväskylä Jyväskylä Finland; ^2^ Department of Teacher Education University of Turku Turku Finland; ^3^ Faculty of Sport and Health Sciences University of Jyväskylä Jyväskylä Finland

**Keywords:** emotional loneliness, family support, ostracism, psychological well‐being, social loneliness, substance use, teacher support

## Abstract

**Introduction:**

This study examined the role of loneliness (social and emotional) and ostracism in adolescents’ psychological well‐being (positive mental health and psychological symptoms) and substance use. Perceived teacher and family support and grade level were examined as moderators in these associations.

**Methods:**

A total of 2241 Finnish adolescents (Grade 7: 1218, *M*_age_ 13.90 years, 50% girls; Grade 9: 1023, *M*_age_ 15.91 years, 52.5% girls) completed a cross‐sectional self‐report survey in 2022. The data were analyzed using structural equation modeling (SEM).

**Results:**

Higher levels of social loneliness and perceived ostracism were associated with poorer psychological well‐being, whereas higher levels of emotional loneliness were associated with frequent substance use. While high teacher support buffered against lower mental health in adolescents with high perceived ostracism, high family support buffered against lower mental health in those with high social loneliness. Higher levels of emotional loneliness were more strongly linked to frequent substance use and lower mental health in older students, whereas the negative association between social loneliness and mental health was stronger in younger students.

**Conclusion:**

These results suggest that it is important to identify the form of social outsiderhood adolescents experience when promoting their well‐being.

## 1 Introduction

The need to belong or feel connected to others is a universal human need (Baumeister and Leary 1995; Ryan and Deci 2020). When unmet, it can lead to social outsiderhood—encompassing loneliness (anxiety that stems from the gap between actual and desired belonging) and ostracism (being ignored and excluded by individuals or groups) (Kiuru et al. 2024; Williams and Nida 2011). Although loneliness and ostracism are related constructs, they arise through different societal interpretations. While loneliness refers to a subjective feeling of unmet social needs, ostracism is related to the actions of others (Kiuru et al. 2024). Social outsiderhood is particularly harmful during adolescence because being accepted in peer groups becomes more important and sensitivity to peer rejection is heightened (Laursen and Hartl 2013; Sebastian et al. 2010). Loneliness has a social (a perceived lack of broader social networks) and an emotional aspect (a perceived lack of close relationships; Weiss 1973); however, little is known about the roles of these aspects in adolescents’ psychological well‐being and substance use. Similarly, research on the role of ostracism in adolescents’ psychological well‐being and substance use is scarce. Following the buffering hypothesis (Cohen and Wills 1985), social support from family and teachers could compensate for low well‐being associated with social outsiderhood. However, there is limited empirical evidence on this topic. Increased understanding of these associations may help develop and target interventions that alleviate social outsiderhood.

### 1.1 The Role of Social Outsiderhood in Adolescents’ Psychological Well‐Being and Substance Use

Adolescence is characterized by several physiological, developmental, and social changes (Eccles and Roeser 2011; Laursen and Hartl 2013). For instance, adolescents seek more autonomy and independence from parents while prioritizing peer relationships (Steinberg and Morris 2001). Consequently, a low sense of belonging to or exclusion from a peer group may increase the risk of social outsiderhood (Beattie et al. 2024; Lynn Mulvey et al. 2017). Here, we examined two separate, yet related, forms of social outsiderhood—loneliness and ostracism.

Loneliness is a multidimensional phenomenon with two aspects: social (a perceived lack of broader social networks) and emotional (a perceived lack of close relationships) (Junttila and Vaura 2009; Weiss 1973). Although these aspects are often correlated, they do not necessarily overlap and can follow quite independent developmental pathways (Maes et al. 2017; Salo et al. 2020). While loneliness is a subjective feeling related to unmet social needs, ostracism is related to the actions of others (Kiuru et al. 2024). Ostracism is a socially painful experience that affects fundamental human needs of belonging, self‐esteem, control, and meaningful existence (Baumeister et al. 2005; Williams and Nida 2011), which if unmet can lead to adverse psychological well‐being (Niu et al. 2016; Wölfer and Scheithauer 2013).

Loneliness may increase vigilance for social threats and make those experiencing it distance themselves from social interactions (Cacioppo and Cacioppo 2018; Spithoven et al. 2017). These cognitive biases and neurobehavioral consequences are further assumed to lead to lower psychological well‐being (Hawkley and Cacioppo 2013). However, studies investigating the roles of the two subtypes of loneliness in psychological well‐being among adolescents are scarce. Of the existing ones, some found emotional loneliness (Wolters et al. 2023) and others found social loneliness to be more strongly related to poorer psychological well‐being (DiTommaso and Spinner 1997; Kiuru et al. 2024). Due to adolescents’ heightened need for acceptance and social belonging (Laursen and Hartl 2013; Sebastian et al. 2010), both forms of loneliness may be particularly harmful in this period and lead to adverse well‐being consequences. Therefore, further knowledge regarding these associations is needed to formulate interventions for the affected adolescents.

Furthermore, social outsiderhood may also act as a risk factor for externalizing problems, including substance use (Hawkley and Cacioppo 2013; Williams 2007). This may be attributed to several reasons; for instance, to overcome the pain caused by and negative effects of social outsiderhood (Khantzian 1997), to gain or maintain social position among peers (Emler and Reicher 1995; Tucker et al. 2011). Previous research has mainly indicated a positive association between loneliness and substance use (Qualter et al. 2013) or an insignificant association between these factors (Dyal and Valente 2015). In turn, Varga and Piko (2015) found a negative association between them, suggesting that lonely adolescents use lesser substances due to the lack of deviant peers. However, one critical limitation of the previous research is that they mainly examined loneliness as a unidimensional construct. Given that social and emotional loneliness are separate constructs with partially different consequences (Maes et al. 2017), investigating further which aspect of loneliness is a more relevant risk factor for substance use is important.

Regarding ostracism, empirical research on its role as a potential risk factor for substance use among adolescents is limited. Sullivan et al. (2006) found that peer exclusion was positively associated with substance use among adolescents. Similarly, Kiuru et al. (2024) found that norm‐breaking behavior and delinquency were typical among ostracized adolescents. Because adolescents are particularly sensitive to peer exclusion (Pharo et al. 2011), more knowledge of this association is needed to help guide interventions for those at risk of heavy substance use.

### 1.2 Family and Teacher Support and Grade Level as Moderators

The relationships between adolescents and their proximal surroundings, such as home and school environments, are crucial for optimal development (Bronfenbrenner 1979). For many adolescents, teachers and family are the main sources of support (Rueger et al. 2016; Virtanen et al. 2022). Following the buffering hypothesis (Cohen and Wills 1985), high support from family and teachers could alleviate or buffer the impact of social outsiderhood‐induced stress thereby increasing their well‐being and decreasing the risk for substance use (Allen et al. 2022; Chu et al. 2010; Zhang et al. 2019). Although there is some empirical evidence showing that supportive relationships may alleviate the negative impact of adolescents’ loneliness on their well‐being (Goodfellow et al. 2022), to our knowledge, no previous study has examined both family and teacher support as moderators in the associations between the two aspects of loneliness and ostracism and psychological well‐being and substance use.

Adolescence involves many changes. Changes such as brain maturation and the development of cognitive abilities may influence how social outsiderhood is interpreted (Laursen and Hartl 2013; Steinberg and Morris 2001). For instance, the ability to regulate emotions including ostracism‐induced distress develops between adolescence and adulthood, suggesting that reactivity to ostracism may be heightened among younger than older adolescents (Sebastian et al. 2010; Young et al. 2019). As cognitive abilities, including abstract reasoning skills and perspective‐taking, become more developed with age (Steinberg 2005), older adolescents may be more aware of and susceptible to the negative impacts of loneliness. In turn, sensation‐seeking and related experimentation with substances are known to increase during adolescence (Steinberg et al. 2018). Moreover, the prevalence of mental health problems and loneliness is higher among older than younger adolescents (Cosma et al. 2023). Therefore, it is possible that the strength of the associations between social outsiderhood and psychological well‐being and substance use varies among adolescents at different ages. However, empirical evidence of these associations is limited. Lyyra et al. (2018) found that the strength of the association between loneliness and psychological symptoms was stronger among older (Grade 9) than younger (Grade 7) students. This study aimed to examine whether the associations between social outsiderhood and psychological well‐being and substance use differ between younger (Grade 7) and older adolescents (Grade 9).

### 1.3 The Present Study

The research questions were as follows:

1. To what extent are the aspects of loneliness (social and emotional) and perceived ostracism related to adolescents’ psychological well‐being (psychological symptoms and positive mental health) and substance use?

Higher levels of social loneliness (DiTommaso and Spinner 1997; Kiuru et al. 2024) and ostracism (Niu et al. 2016; Sebastian et al. 2010) are related to more frequent psychological symptoms, whereas higher levels of emotional loneliness and ostracism are related to more frequent substance use (Kiuru et al. 2024; Sullivan et al. 2006). Due to limited research, no hypotheses for the roles of these aspects in positive mental health were set.

2. To what extent does social support from family and teachers moderate the associations between social outsiderhood and adolescents’ psychological well‐being and substance use?

High teacher and family support buffers against more frequent psychological symptoms and substance use for adolescents experiencing high social and emotional loneliness and ostracism (Allen et al. 2022; Baumeister and Leary 1995; Goodfellow et al. 2022; Zhang et al. 2019).

3. To what extent does grade level (Grade 7 vs. Grade 9) moderate the associations between social outsiderhood and adolescents’ psychological well‐being and substance use?

Due to the lack of studies regarding social and emotional loneliness, no exact hypotheses could be set. Nevertheless, we expect that loneliness is more strongly related to psychological symptoms and substance use in Grade 9 than in Grade 7 students (Lyyra et al. 2018; Steinberg et al. 2018), and ostracism is more strongly related to psychological symptoms and substance use in Grade 7 than in Grade 9 students (Sebastian et al. 2010).

## 2 Materials and Methods

### 2.1 Participants

Data for this study were obtained from the 2022 Finnish Health Behavior in School‐aged Children (HBSC) survey. The HBSC is an international World Health Organization collaborative study where students aged 11, 13, and 15 complete questionnaires anonymously every 4 years. Participation was voluntary. Before data collection, study information was sent to parents, giving them the opportunity to opt out if they did not want their adolescents to participate. The HBSC study was conducted according to the Declaration of Helsinki. The questionnaire was approved by the Ethics Committee of the local university.

The included adolescents were in lower secondary school. Schools were selected from the Finnish school register using random cluster sampling, with the school as the primary sampling unit. In each school, one class was randomly selected from the relevant grade. Sampling followed the international HBSC protocol (Inchley et al. 2018; Schnohr et al. 2015) to ensure a nationally representative sample. Overall, the study included 2241 adolescents (50.6% girls): Grade 7—1218, *M*_age_ 13.90 years, SD = 0.42, and 50% girls, and Grade 9—1023, *M*_age_ 15.91 years, SD = 0.42, and 52.5% girls. Of these, 62.1% lived in nuclear families, 10.3% in stepfamilies, 14.8% in single‐parent families, 8.5% in foster homes or with someone else (e.g., grandparents), and 4.3% provided no information about their family structure.

### 2.2 Measures

The descriptive statistics and correlations between the main study variables are presented in Table 1.

**Table 1:** Descriptive statistics and pearson correlations between the main study variables.

		*n*	*M*	SD	Min–Max	1	2	3	4	5	6	7	8	9	10	11	12	13	14	15	16	17
1	Social loneliness	2193	2.28	0.93	1–5																	
2	Emotional loneliness	2164	1.81	1.08	1–5	0.59***																
3	Ostracism	2179	2.20	0.98	1–5	0.79***	0.57***															
4	Positive mental health	2138	24.35	5.42	7–35	–0.46***	–0.36***	–0.44***														
5	Psychological symptoms Feeling low	2212	2.52	1.36	1–5	0.48***	0.27***	0.47***	–0.52***													
6	Irritability	2213	2.98	1.24	1–5	0.35***	0.19***	0.34***	–0.41***	0.65***												
7	Feeling nervous	2213	2.81	1.28	1–5	0.37***	0.19***	.38***	–0.42***	0.64***	0.66***											
8	Feeling tension	2208	2.67	1.23	1–5	0.36***	0.22***	0.37***	–0.38***	0.59***	0.56***	0.76***										
9	Anxiety	2205	2.43	1.45	1–5	0.47***	0.29***	0.46***	–0.51***	0.75***	0.59***	0.70***	0.68***									
10	Difficulties in getting to sleep	2214	2.47	1.38	1–5	0.32***	0.23***	0.33***	–0.39***	0.50***	0.45***	0.46***	0.43***	0.49***								
11	Waking up at night	2200	2.10	1.30	1–5	0.27***	0.19***	0.27***	–0.33***	0.39***	0.35***	0.34***	0.33***	0.40***	0.55***							
12	Substance use Smoking cigarettes	2090	1.43	1.29	1–7	0.11***	0.17***	0.12***	–0.16***	0.09***	0.06**	0.04*	0.05*	0.10***	0.11***	0.12***						
13	E‐cigarette use	2098	1.38	1.23	1–7	0.15***	0.19***	0.15***	–0.17***	0.06**	0.05*	0.03	0.03	0.08***	0.11***	0.13***	.64***					
14	Drinking alcohol	2079	1.39	1.06	1–7	0.17***	0.21***	0.16***	–0.19***	0.11***	0.09***	0.08***	0.08***	0.12***	0.14***	0.16***	.61***	.64***				
15	Family support	1999	5.44	1.67	1–7	–0.35***	–0.36***	–0.35***	0.49***	–0.34***	–0.27***	–0.26***	–0.23***	–0.36***	–0.27***	–0.23***	–0.20***	–0.20***	–0.23***			
16	Teacher support	2053	3.80	0.94	1–5	–0.34***	–0.29***	–0.36***	0.43***	–0.30***	–0.24***	–0.22***	–0.21***	–0.30***	–0.24***	–0.19***	–0.20***	–0.21***	–0.22***	.32***		
17	Grade level (0 = Grade 7, 1 = Grade 9)	2241			0–1	0.08***	0.04*	0.07***	0.01	0.05*	–0.03	–0.01	0.01	0.07***	–0.01	0.02	.12***	.07***	.11***	–0.07**	–0.02	

*** *p* < 0.001

** *p* < 0.01

* *p* < 0.05

*Social loneliness* was measured using two questions: “How often have you felt lonely over the past 12 months?” (e.g., Eccles et al. 2020) and “During the past 12 months, how often have you felt that you lack companionship?” (Roberts et al. 1993). The questions were answered on a 5‐point scale (1 = never, 2 = rarely, 3 = sometimes, 4 = most of the time, 5 = always; α = 0.72). Short loneliness scales (e.g., UCLA‐4 and direct single‐item measure of loneliness) are widely used in surveys and show good concurrent and predictive validity (Eccles et al. 2020; Mund et al. 2023).

*Emotional loneliness* was measured using a single question derived from the UCLA‐4 scale: “During the past 12 months, how often have you felt that you no longer have anyone close to you?” (Eccles et al. 2020; Roberts et al. 1993). Students responded to the question using a 5‐point scale (1 = never, 5 = always).

*Ostracism* was measured using two questions related to subjective experience of social exclusion derived from the UCLA‐4 scale: “During the past 12 months, how often have you felt left out? and “…how often have you felt isolated from others? (Eccles et al. 2020; Roberts et al. 1993). Students rated these questions on a 5‐point scale (1 = never, 5 = always; *α* = 0.87).

*Positive mental health* was measured using a short version of the Warwick‐Edinburgh Mental Well‐being Scale (Tennant et al. 2007), which consists of seven items (e.g., “I have been feeling optimistic about the future”) and measured on a 5‐point scale (1 = never, 5 = always; *α* = 0.91).

*Psychological symptoms* were assessed using the nonclinical measures of health complaints designed for the HBSC study (Inchley et al. 2022). Students were asked how often they experienced the following in the last 6 months: feeling low, irritability or bad temper, feeling nervous, feeling tension, anxiety, difficulties in getting to sleep, and waking up at night. The items were answered using a 5‐point scale (1 = about every day, 2 = more than once a week, 3 = about every week, 4 = about every month, 5 = rarely/never; *α* = 0.89). The items were reversed so that higher scores represent more frequent psychological symptoms.

*Substance use* was measured using three questions: “In the last 30 days, on how many days (if any) have you (i) smoked cigarettes, (ii) used e‐cigarettes, and (iii) drank alcohol?” (Inchley et al. 2022). Students answered these questions on a 7‐point scale (1 = never, 2 = 1–2 days, 3 = 3–5 days, 4 = 6–9 days, 5 = 10–19 days, 6 = 20–29 days, 7 = 30 days or more; *α* = 0.83).

*Social support from family* was assessed using the Multidimensional Scale of Perceived Social Support (Inchley et al. 2022; Zimet et al. 1988), which consists of four items (e.g., “I get the emotional help and support I need from my family”; *α* = 0.95) and measured on a 7‐point scale (1 = strongly disagree, 7 = strongly agree).

*Social support from teachers* was measured using the Teacher Support Scale (Inchley et al. 2022; Torsheim et al. 2000), which consists of three items (e.g., “I feel that my teachers care about me as a person”; *α* = 0.87) on a 5‐point scale (1 = strongly agree, 5 = strongly disagree). The items were reversed so that higher scores indicate higher teacher support.

Adolescent biological sex (0 = boy, 1 = girl) and socioeconomic status (SES) were used as control variables because girls report higher social loneliness, and boys experience higher emotional loneliness (Salo et al. 2020), and lower SES is linked to higher loneliness (Algren et al. 2020). SES was assessed using the Family Affluence Scale III (FAS‐III; Currie et al. 2024), which includes six questions on family wealth (e.g., “How many computers, laptops, and tablets, not including game consoles and smartphones, does your family own?). An aggregated FAS index (range, 0–13) was calculated. If two or more questions were unanswered, the FAS index was not calculated.

### 2.3 Analytical Strategy

The analyses were executed in the following order. First, measurement models for adolescents’ psychological symptoms, mental health, and substance use were estimated separately using confirmatory factor analysis (CFA). Next, SEM was utilized to estimate the paths from the aspects of loneliness (social and emotional) and ostracism to psychological symptoms, mental health, and substance use while controlling for the effects of adolescent sex and SES. In the SEM model, latent factors were allowed to correlate with each other, as well as the residuals of the observed variables.

Second, CFA was utilized to estimate measurement models for family and teacher support. In the CFA, factors were allowed to correlate. To examine the moderating effects of family and teacher support, a total of six latent interaction terms were defined and added to the previous SEM model: 2 for social loneliness (social loneliness × teacher support and social loneliness × family support), 2 for emotional loneliness (emotional loneliness × teacher support and emotional loneliness × family support), and 2 for ostracism (ostracism × teacher support and ostracism × family support). Before defining the interaction terms, the independent variables (social and emotional loneliness and ostracism) were mean‐centered, and moderator factors were standardized.

Finally, a multigroup approach was utilized to examine whether the associations between the aspects of loneliness and ostracism and psychological well‐being and substance use vary between Grade 7 and Grade 9 adolescents. Wald test was used to determine whether the same model was suitable for both grade levels. The model constraint command was further used to test the possible differences between Grade 7 and Grade 9 students in the associations between the observed variables and latent outcome variables.

The analyses were performed using the COMPLEX approach of Mplus (Version 8.11; Muthén and Muthén 1998–2025) to consider the clustered nature of the data. The proportion of missing data for the main study variables ranged from 2.1% to 10.8% (*M* = 5.52, SD = 2.63). The models were estimated using FIML with MLR. The goodness‐of‐fit of the models was evaluated using the following fit indicators: *χ* ^*2*^ test, comparative fit index (CFI), root mean square error of approximation (RMSEA), and standardized root mean square residual (SRMR). Nonsignificant *χ* ^*2*^ values, CFI > 0.95, RMSEA < 0.06, and SRMR < 0.08 were considered indicators of good model fit (Hu and Bentler 1999).

## 3 Results

### 3.1 Associations between Social Outsiderhood and Psychological Well‐Being and Substance Use

First, CFA was utilized to construct measurement models for adolescents’ psychological symptoms, mental health, and substance use. After allowing two residual correlations between items measuring psychological symptoms—feeling tension and nervous (*standardized estimate of freed residual correlation* =.41, SE = 0.03, *p* <0.001) and difficulties in getting to sleep and waking up at night (*standardized estimate of freed residual correlation* = 0.39, SE = 0.02, *p* < 0.001), the measurement models fit the data well: *χ* ^2^[3–14] = 101.33–393.41, *p*s < 0.001, CFIs = 0.98–1.00, RMSEAs = 0.00–0.07, and SRMRs = 0.00–0.02.

**Figure 1:**
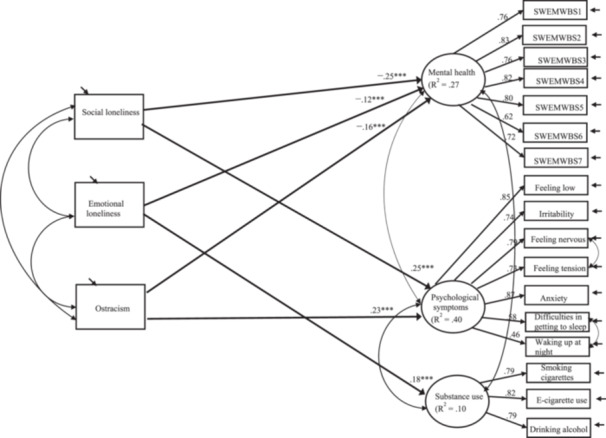
Associations between the aspects of loneliness and ostracism and psychological well‐being and substance use. *Note:* Fit of the model: *χ* ^2^ [183] = 852.77, *p* < 0.001, CFI = 0.96, RMSEA = 0.04, and SRMR = 0.04. Standardized estimates are presented. Only statistically significant paths are shown. ***p < 0.001.

Next, SEM was utilized to answer the first research question. The results are presented in Figure 1. The results showed that higher levels of social and emotional loneliness and ostracism were associated with lower mental health, and higher levels of social loneliness and ostracism were associated with more frequent psychological symptoms. Only emotional loneliness was related to substance use; the more emotional loneliness adolescents experienced, the more they used substances. Due to cross‐sectional data, causal direction cannot be determined.

### 3.2 Support From Teachers and Family As Moderators

**Figure 2:**
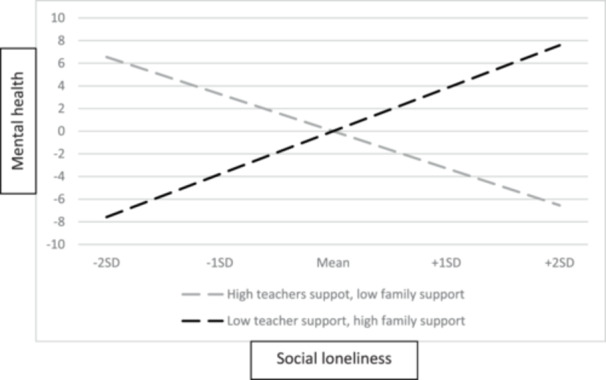
Teacher and family support as moderators between adolescent social loneliness and mental health.

First, CFA was utilized to create a measurement model for teacher and family support. The model fit was good: *χ* ^2^ [13] = 50.93, *p* < 0.001, CFI = 0.99, RMSEA = 0.04, and SRMR = 0.02. Second, a total of six interaction terms tapping the relationship between teacher support and observed predictors (social and emotional loneliness and ostracism) and family support and observed predictors were defined and added to the previous SEM model. The results showed that the interaction term social loneliness × teacher support was associated with mental health (*unstandardized estimate* = −1.66, SE = 0.60, *p* < 0.01, 95% CI [−2.85, −0.48]). Similarly, the interaction term social loneliness × family support was associated with mental health (*unstandardized estimate* = 1.87, SE = 0.53, *p* < 0.001, 95% CI [0.84, 2.90]). To investigate the significant interaction effects more closely, the simple slopes for low (−1 SD) and high (+1 SD) levels of both teacher and family support were calculated. Four combinations of simple slopes (high teacher support‐high family support, low teacher support‐low family support, high teacher support‐low family support, and low teacher support‐high family support) were defined (see Stride et al. 2015). Only statistically significant combinations of simple slopes are presented. The results are demonstrated in Figure 2. When teacher support was low but family support was high, higher social loneliness was related to higher mental health (*unstandardized estimate* = 3.79, *SE* = 0.95, *p* < 0.001). This result suggests that in the absence of teacher support, supportive home environment may help to maintain positive mental health for adolescents experiencing high social loneliness.

**Figure 3:**
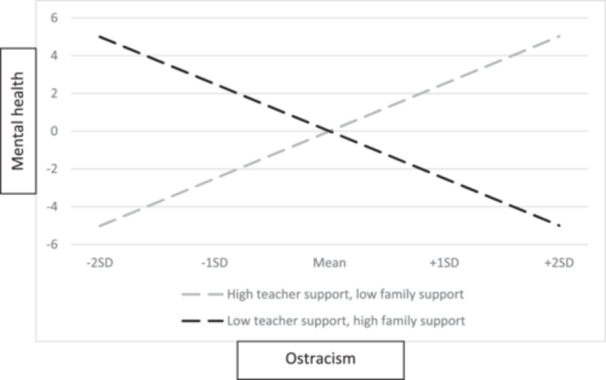
Teacher and family support as moderators between adolescent ostracism and mental health.

The results also showed that the interaction term ostracism × teacher support was associated with mental health (*unstandardized estimate* = 1.15, SE = 0.31, *p* < 0.001, 95% CI [0.54, 1.75]). Similarly, the interaction term ostracism × family support was associated with mental health (*unstandardized estimate* = −1.36, SE = 0.32, *p* < 0.001, 95% CI [ − 1.99, −0.74]). The interaction is demonstrated in Figure 3. When teacher support was high but family support was low, higher ostracism was related to higher mental health (*unstandardized estimate* = 2.51, SE = 0.74, *p* < 0.01). When teacher support was low but family support was high, higher ostracism was related to lower mental health (*unstandardized estimate* = −2.50, SE = 0.77, *p* < 0.01). These results indicate that among adolescents who experience high levels of ostracism, a supportive home environment may not be sufficient to maintain positive mental health if they perceive a lack of support from teachers.

### 3.3 Grade Level as a Moderator

**Figure 4:**
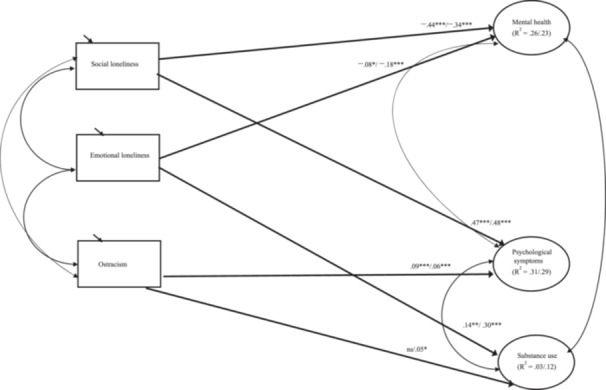
Associations between the aspects of loneliness and ostracism and psychological well‐being and substance use. *Note:* Fit of the model: *χ* ^2^ [408] = 1468.59, *p* < 0.001, CFI = 0.94, RMSEA = 0.05, and SRMR = 0.06. Standardized estimates are presented. The first coefficient is for Grade 7 students (*n* = 1214) and the second coefficient is for Grade 9 students (*n* = 1023). ***p < 0.001; **p < 0.01; *p < 0.05.

To answer the third research question, multigroup SEM was utilized. The results of the Wald test showed that parameter constraints were not equal between the two grade levels: *W* (9) = 20.57, *p* < 0.05 (see Figure 4). First, the association between social loneliness and mental health was different between Grade 7 and Grade 9 students *(unstandardized estimate* = –0.10, SE = 0.05, *p* < 0.05). Social loneliness was more strongly associated with mental health in Grade 7 than in Grade 9 students. Second, the association between emotional loneliness and mental health was different between Grade 7 and Grade 9 students (*unstandardized estimate* = 0.07, SE = 0.04, *p* < 0.05). Emotional loneliness was more strongly related to mental health in Grade 9 than in Grade 7 students.

Similarly, the results showed that the association between emotional loneliness and substance use was different between Grade 7 and Grade 9 students (*unstandardized estimate* = –0.16, SE = 0.06, *p* < 0.01). Emotional loneliness was more strongly related to substance use in Grade 9 than in Grade 7 students. Other significant differences were not found between Grade 7 and Grade 9 students.

## 4 Discussion

This study examined the roles of social and emotional loneliness and perceived ostracism in psychological well‐being and substance use among adolescents, with support from teachers and family and grade level as moderators of these associations. The results revealed that higher levels of social loneliness and ostracism were associated with lower mental health and more frequent psychological symptoms. Higher levels of emotional loneliness were associated with poorer mental health and more frequent substance use. Teacher and family support acted as buffers in the associations between social outsiderhood and mental health. Furthermore, emotional loneliness was more strongly related to positive mental health and substance use in older students, whereas social loneliness was more strongly related to positive mental health in younger students. These results underline the importance of identifying the type of social outsiderhood adolescents experience to effectively promote their well‐being and prevent psychological problems.

Partly in line with our expectations and previous studies (Lynn Mulvey et al. 2017; Niu et al. 2016), higher levels of ostracism were related to more frequent psychological symptoms and lower mental health. These results suggest that being excluded and/or ignored by peers thwart fundamental human needs (Williams and Nida 2011; Wölfer and Scheithauer 2013), negatively affecting psychological well‐being. Surprisingly, ostracism was not related to substance use. This may be because, in contrast to previous studies (Kiuru et al. 2024; Sullivan et al. 2006), we did not consider other types of externalizing problems (e.g., delinquency and conduct problems). Therefore, future studies should implement broader assessments to better understand the role of ostracism in adolescents’ externalizing problems. It may also be that ostracism is related to substance use via psychological distress (Williams 2007). This indirect pathway should be investigated in future research.

In accordance with our expectations and some previous studies (DiTommaso and Spinner 1997; Kiuru et al. 2024), the results showed that higher levels of social loneliness were related to more frequent psychological symptoms and lower mental health. This suggests that lacking social networks can create cognitive biases that hinder lonely adolescents’ social functioning (Hawkley & Cacioppo 2013; Spithoven et al. 2017) and decrease a sense of belonging, leading to psychological ill‐being. As expected, higher levels of emotional loneliness were associated with more frequent substance use, suggesting that substance use is a way of coping for adolescents longing for close relationships (Khantzian 1997) or increasing popularity among peers (Tucker et al. 2011). Because peers are important sources of support during adolescence (Letkiewicz et al. 2023), substance use may serve as a coping response to a lack of friend support. However, given the correlational nature of our study, the possibility that adolescents who use substances and suffer from psychological ill‐being are more prone to distance themselves from others and thus experience higher levels of loneliness exists (Chen et al. 2023). Future longitudinal studies are needed to examine the possible reciprocal associations between the aspects of loneliness and psychological well‐being and substance use.

In contrast to our expectations, high teacher or family support did not buffer against psychological ill‐being but instead helped maintain positive mental health. However, different result patterns were observed depending on the type of social outsiderhood and the source of support. When teacher support was lacking, high family support helped to maintain positive mental health for adolescents perceiving high social loneliness. In turn, the results showed that among students who felt ostracized, support from family was not sufficient to maintain their positive mental health if they perceived a lack of support from teachers. Since school is a vital social context in adolescence (Letkiewicz et al. 2023), teacher support may become essential because it helps to ease the social pain caused by perceived peer exclusion (Laursen and Hartl 2013; Williams and Nida 2011). Overall, these results highlight the importance of identifying the form of social outsiderhood adolescents experience when maintaining and promoting their mental health.

Regarding the moderating role of grade level, partly in line with the hypothesis and previous research (Lyyra et al. 2018), our results showed that emotional loneliness was more strongly related to poorer mental health and more frequent substance use in older (Grade 9) than in younger (Grade 7) students. Sensation‐seeking and related experimentation with substances increase throughout adolescence (Steinberg et al. 2018). As older adolescents’ cognitive abilities—such as abstract reasoning skills and the capacity for perspective‐taking—become more developed (Steinberg 2005), they may also experience heightened awareness and sensitivity to emotional loneliness, which, in turn, lead them to use more substances. However, contrary to our expectations, the negative link between social loneliness and mental health was stronger among younger than older students. It should be noted that, on average, adolescents in Grade 9 experienced more social and emotional loneliness and ostracism than younger adolescents. The results suggest that there are age‐related differences in how strongly the experiences of social outsiderhood are related to mental health and substance use. As emotion regulation skills develop throughout adolescence (Young et al. 2019), younger students may have less developed emotion regulation skills to cope with social loneliness. However, it should be noted that the age difference between grade levels was relatively small (Grade 7, *M*_age_ 13.90 years; Grade 9, *M*_age_ 15.91 years). More research is needed to understand how social outsiderhood is interpreted across different age groups to formulate age‐appropriate interventions.

## 5 Limitations and Future Directions

This study has certain limitations. First, the aspects of social outsiderhood were measured using short scales. However, there is an indication that shorter loneliness scales could be valid in epidemiologic studies (Eccles et al. 2020; Roberts et al. 1993), which provides some confidence in the results of this study. Moreover, the timeframe of the items used to measure social outsiderhood was relatively long (12 months) and thus, may include recall bias. Second, this study was cross‐sectional, making causal inferences challenging to determine. Following syndemics theory (Singer 1996), the aspects of social outsiderhood should not be viewed as isolated risk factors but rather interactive conditions that collectively undermine psychological ill‐being and increase the risk of substance use. In subsequent studies, examining the co‐trajectories and interactions among these aspects could provide multifaceted information into how their cumulative effects contribute to adolescents’ psychological well‐being and the risk of substance use in the long term. Third, even though nicotine pouches have increased in popularity among adolescents (Ruokolainen et al. 2024), we did not have information on the frequency of nicotine pouch use. Thus, future studies should include more comprehensive measures of substance use to better understand their antecedents and consequences. Given that substances have different social meanings (Kobus and Henry 2010), in subsequent studies, it would be fruitful to examine the relationship between social outsiderhood and each substance separately. Fourth, gender was included as a control variable. Given the found gender differences in loneliness (Maes et al. 2017; Salo et al. 2020) and in the strength of the associations between loneliness and psychological well‐being (Lyyra et al. 2018), future research could investigate whether the moderating effects of teacher and family support on these associations differ by gender. Finally, the data were collected post‐COVID pandemic. There is research showing that adolescent‐perceived loneliness is more strongly linked to mental health problems in post‐pandemic samples than in pre‐pandemic ones (Gustafsson et al. 2023). This suggests the need to promote resilience during exceptional circumstances.

## 6 Conclusion

Social and emotional loneliness entailed different problems. Due to emotional loneliness being a more significant predictor of lower mental health and more frequent substance use among older students and social loneliness being a stronger predictor of lower mental health among younger students, it is important to consider adolescents’ developmental stages when planning interventions. Moreover, because teachers have important roles in compensating for lower mental health associated with high ostracism, more knowledge and tools should be made available to them to identify ostracized students. Evidence‐based social‐emotional learning programs that promote self‐awareness, self‐management, and social skills may be useful for reducing psychological problems and social exclusion in school context (Fernández‐Martín et al. 2024). More awareness and education for parents on how they can support lonely adolescents could also be beneficial. Parents should encourage open communication and trust in their relationships with their adolescents (Madsen et al. 2025). Given that parents’ psychological problems and loneliness may negatively affect their parenting which, in turn, influence adolescents’ well‐being (Nowland et al. 2021), interventions aimed at improving adolescents’ well‐being and alleviating social outsiderhood should also address parental mental health and social support. For adolescents, promoting social skills and emotion regulation and providing opportunities to practice these skills could be used to alleviate loneliness (Turner et al. 2024), whereas interventions focusing on peer relationships (Lei et al. 2024) and resilience (Niu et al. 2016) may be useful to decrease ostracism.

## Ethics Statement

This study, including the questionnaire, was approved by the Ethics Committee of the local university. The HBSC study was conducted in accordance with the Declaration of Helsinki.

## Consent

All participants provided informed consent. The guardians were informed of the study and given the opportunity to decide their child's ( < 15 years) participation in the research.

## Conflicts of Interest

The authors declare no conflicts of interest.

## Data Availability

The 2022 HBSC data will be made available in the Data Management Centre repository (https://www.uib.no/en/ hbscdata/113290/open‐access) in 2026, following HBSC protocol.
